# Additive and Photochemical Manufacturing of Copper

**DOI:** 10.1038/srep39584

**Published:** 2016-12-21

**Authors:** Winco K. C. Yung, Bo Sun, Zhengong Meng, Junfeng Huang, Yingdi Jin, Hang Shan Choy, Zhixiang Cai, Guijun Li, Cheuk Lam Ho, Jinlong Yang, Wai Yeung Wong

**Affiliations:** 1 Department of Industrial and Systems Engineering, The Hong Kong Polytechnic University, Hung Hom, Hong Kong; 2 School of Reliability and Systems Engineering, Beihang University, No. 37 Xueyuan RD. Haidian, Beijing 100191, China; 3 Institute of Molecular Functional Materials and Department of Chemistry, Hong Kong Baptist University, Waterloo Road, Hong Kong; 4 Hefei National Laboratory for Physical Sciences at Microscale, University of Science and Technology of China, Hefei, Anhui 230026, China; 5 Department of Applied Biology and Chemical Technology, The Hong Kong Polytechnic University, Hung Hom, Hong Kong

## Abstract

In recent years, 3D printing technologies have been extensively developed, enabling rapid prototyping from a conceptual design to an actual product. However, additive manufacturing of metals in the existing technologies is still cost-intensive and time-consuming. Herein a novel platform for low-cost additive manufacturing is introduced by simultaneously combining the laser-induced forward transfer (LIFT) method with photochemical reaction. Using acrylonitrile butadiene styrene (ABS) polymer as the sacrificial layer, sufficient ejection momentum can be generated in the LIFT method. A low-cost continuous wave (CW) laser diode at 405 nm was utilized and proved to be able to transfer the photochemically synthesized copper onto the target substrate. The wavelength-dependent photochemical behaviour in the LIFT method was verified and characterized by both theoretical and experimental studies compared to 1064 nm fiber laser. The conductivity of the synthesized copper patterns could be enhanced using post electroless plating while retaining the designed pattern shapes. Prototypes of electronic circuits were accordingly built and demonstrated for powering up LEDs. Apart from pristine PDMS materials with low surface energies, the proposed method can simultaneously perform laser-induced forward transfer and photochemical synthesis of metals, starting from their metal oxide forms, onto various target substrates such as polyimide, glass and thermoplastics.

Emerging additive manufacturing of metals is being intensively studied for building drop-on-demand electronics by computer-aided design^1^,^2^; selective laser sintering^3^ and inkjet printing^4^,^5^ strategies have been widely investigated to overcome their drawbacks. Selective laser sintering involves heating the metal to its melting point, which is both energy-intensive and time-consuming. Meanwhile, inkjet printing suffers from clogging issues, in which the nano-sized ink has to be overcome. Therefore, the mass production of electrical circuits, electrodes, and antennas with high production yields and low raw material costs is still challenging in current additive manufacturing^6^.

Laser-induced Forward Transfer (LIFT) is an alternative additive manufacturing advancement for directly transferring the desired materials onto a target substrate^7^. The mechanisms involved in using a pulsed laser for LIFT of metals have been comprehensively studied^8^,^9^,^10^. The traditional non-contact LIFT using the pulsed laser has three steps, as represented in Fig. S1(a,b,d). The acceptor layer, donor layer and transparent carrier are illustrated in Fig. S1(a) from bottom to top, respectively. After the pulsed laser spot is focused on the donor layer, the illuminated zone starts to increase its volume due to thermal expansion of the donor layer material, as displayed in Fig. S1(b). With the optimized pulsed laser power and acceptor-to-donor distance, the ejected droplet can eventually be transferred onto a donor layer, as revealed in Fig. S1(d). Attributed to the development of laser techniques, the LIFT method is a rapid platform for the deposition of functional materials. Taking advantage of the thermal expansion of materials, the LIFT method saves time and energy in comparison with selective laser sintering, which usually needs significantly high temperatures^11^. A handful of metals, such as gold^2^,^12^, silver^13^, copper^2^, aluminum^14^, and chromium^15^,^16^, have been deposited using this LIFT method. Various types of applications including emerging energy devices and advanced 3D structures have also been utilized using the LIFT techniques^17^,^18^,^19^,^20^,^21^,^22^,^23^. Using vacuum deposition methods, such as sputtering^24^ and thermal evaporation^25^, pure thin metal films were first grown on transparent carrier substrates. However, vacuum deposition methods are usually cost-intensive and time-consuming^26^. In contrast, solution based methods are more efficient for preparing donor films on carrier substrates at low cost^27^,^28^. For example, 15 μm thick LiCoO_2_ films were coated on glass substrates using a wire coater as the donor layer for LIFT^29^. However, it is challenging to directly apply thick pure metal layers onto the carrier layer using wet methods. Besides, nano-sized metals can easily be oxidized in wet solutions because the ratios of their surface areas to volume are usually high^30^. In addition, the cost of the pulsed laser systems is usually rather expensive for general use.

Similar to synthesizing pure metals from their oxide minerals, most metals can be chemically reduced from their metal oxides^31^, even using photochemical mechanisms. The mechanisms of this light induced reduction are typically based on photo-thermal and homolysis effects. The emerging intense pulsed light has been successfully demonstrated in reducing copper oxide to metallic copper through local heating with pre-defined patterns^32^. The other photochemical mechanism for synthesizing metal is through homolysis, usually with a UV light source. The minimum energy for homolysis is defined as the bond dissociation energy. The resistivity and the bond dissociated energy of the commonly used metal are summarized and listed in Table S1^33^. Silver possesses the lowest resistivity among all metals and its bond dissociation energy is only 221 kJ/mol, which corresponds to the photon energy at a wavelength of 541 nm. However, the price of silver, significantly, is too high for mass production. In comparison, copper possesses the second lowest resistivity with bond dissociation energy of 287.4 kJ/mol, corresponding to a photon at the wavelength of 416 nm. Even the (Al,In)GaN laser diode at 405 nm, with a price below 50 USD, would be able to trigger the homolysis of copper oxide, in theory. Besides, the raw material cost of copper oxide is also significantly lower than that of silver. Hence copper is an optimized candidate for synthesis through homolysis in respect to resistivity, bond dissociation energy and the raw material cost.

The ejection momentum is the most important factor for transferring the target material from the donor layer to the acceptor layer in LIFT^34^. A sacrificial layer, called the dynamic release layer, is usually applied between the carrier and the donor layer to increase the momentum during LIFT. The deformation of the film due to thermal expansion can escape the surface tension and fly away as droplets^34^,^35^,^36^. For example, the Laser-induced Thermal Imaging (LITI) method acquires sufficient ejection momentum using the thermally sacrificial layer^37^. In order to facilitate silver nanoparticle ink in LIFT, an inorganic titanium layer with relatively low melting temperature was used as the sacrificial layer^38^. Meanwhile, the organic triazene polymer with low decomposition temperature was used to perform the LIFT of GdGaO for LED applications^39^. For the LIFT of metals, an organic sacrificial layer is better than a metallic one, because of its relatively low sublimation and boiling temperature, and the absence of metal contamination. Among the commercialized organic thermoplastics, ABS polymer is widely used due to its low cost. It has a thermal expansion of 73.8 × 10^−6^ K^−1^ and a relatively low decomposition temperature of 400 °C. Its chemically-active decomposition product would react with O_2_ under ambient conditions, giving out CO_2_ in the gas phase and further increasing the volume expansion^40^. The generated gas promotes volume expansion and improves the ejection momentum for the donor layer material, as sketched in Fig. S1(c). The ABS polymer was thus chosen as a sacrificial layer to increase the ejection momentum during LIFT in this work^34^.

By combining the photochemically reducible copper oxide with the sacrificial ABS polymer, a novel LIFT method is proposed and investigated here. The *ab initio* calculation of CuO suggested that the optical absorption coefficient was sufficiently high at 405 nm, so the low cost (Al,In)GaN laser diode with output power fixed at 0.4 W was used to undertake the LIFT. The CuO and ABS powders were mechanically mixed in chloroform, and then glued together after solvent drying. The microstructures, chemical composites and the crystalline structures of the CuO/ABS before and after LIFT were experimentally studied, and it was shown that significant amounts of copper were synthesized after the laser treatment. Post electroless plating processes were also performed to lower the surface roughness and resistivity of the synthesized copper. Prototypes of circuit interconnections were demonstrated to power up LEDs and the resistivity of the circuits was measured to be as low as 1.29 × 10^−7^ Ωm, which is only 7 times larger than that of the bulk copper. The ease of patterning copper on various substrates using this LIFT method depends on the surface energy, which was verified by water contact angle measurements.

## Results and Discussion

**Figure 1:**
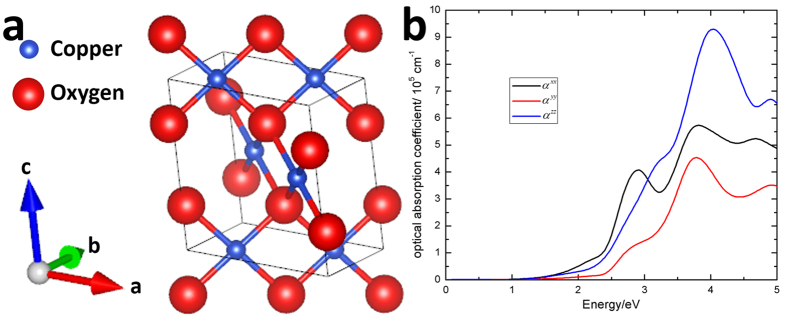
Ionic structure and optical absorption coefficient of CuO. (**a**) Molecule structure of CuO. (**b**) Optical absorption coefficient according to photon energy calculated from DFT simulation.

The ionic structures of bulk CuO are illustrated in Fig. 1(a), where the copper atoms and the oxygen atoms are denoted as small blue spheres and large red spheres respectively. The copper(II) oxide appears in monoclinic crystalline structures, and the lattice constants optimized by PBE are a = 4.653 Å, b = 3.410 Å, c = 5.108 Å and β = 99.48°, matching well with the experimental values (a = 4.684 Å, b = 3.423 Å, c = 5.129 Å and β = 99.54°). The optical absorption coefficients of copper(II) oxide according to different photon energies were calculated with the DFT method and are shown in Fig. 1(b). According to the Stark-Einstein law, only one molecule would be activated in the following reaction with each absorbed photon. So the photon energy corresponding to the laser wavelength is important for the homolysis reaction. In the DFT calculation results, the optical absorption coefficients near a photon energy of 3.06 eV (exact value at 3.059 eV in DFT calculation) are 3.68 × 10^5^ cm^−1^, 1.52 × 10^5^ cm^−1^, 3.77 × 10^5^ cm^−1^, in the xx, yy, and zz directions, respectively. Thus the optical absorption is relatively high at 405 nm, allowable for triggering the LIFT process according to Grotthuss-Draper law^41^,^42^.

**Figure 2:**
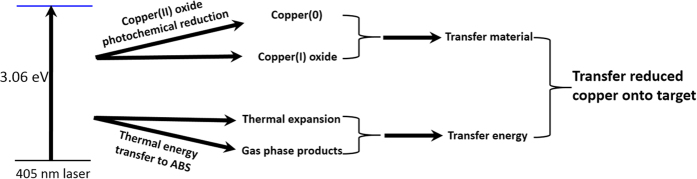
Process diagram of the LIFT with CuO/ABS composition in the forms of different energies.

The overall mechanism of this novel LIFT process is depicted in Fig. 2 according to the two species of energies involved, i.e., photochemical and thermal energies. After the 405 nm laser illumination, the CuO can effectively absorb the photon energies as proven in the DFT results. Through the homolysis process, the copper(II) oxide dissociates into copper and copper(I) oxide, and the photochemical products would then be the materials transferred to the donor layer during the LIFT. Meanwhile, the ABS polymer absorbs the photon energy in the form of heat. The thermal energy results in the decomposition and thermal expansion on the ABS polymers. The increased volume from both the decomposition and the thermal expansion of the ABS polymers produces the momentum for the transferred materials, completing the donor to acceptor LIFT process. The ejection process is similar to LITI, except that the sacrificial layer here is mixed with the copper oxide as the copper precursor.

**Figure 3:**
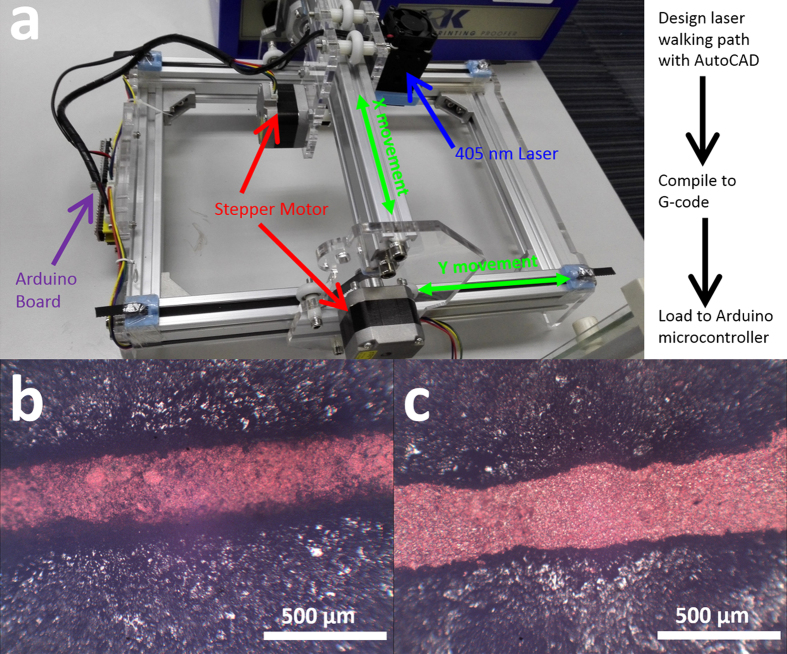
Laser processing system and the optical microscope images for the samples. (**a**) The photo of a tailor-made laser writing system. (**b**) The optical microscope image of a single trace of Cu as patterned by LIFT. (**c**) The optical microscope image of the single trace after post electroless copper plating.

The optical microscope image of the as-transferred circuit line is shown in Fig. 3(b). Appearing with random black dots, the bronze color line pattern is approximately 200 μm wide. The height of this pattern is 13.67 μm, as determined by the stylus profilometer. The resistivity of the as-prepared sample is 1.46 × 10^−6^ Ωm as revealed by standard four-probe measurement, and is 87 times larger than that of the bulk copper (1.68 × 10^−8^ Ωm). The as-LIFT copper using this low-cost method did not have satisfactory conductivity for direct application as compared to the recent strike with pulsed lasers^2^. To improve its electrical performance, industry-graded electroless plating was performed on the as-prepared sample to further lessen its resistivity^43^. An optical microscope image of the circuit line after electroless-plating is shown in Fig. 3(c). In comparison with the as-prepared sample in Fig. 3(b), the line pattern after the post electroless-plating is brighter and has fewer black dots. The height of the plated line is increased to 20.54 μm, and the resistivity is decreased to 1.29 × 10^−7^ Ωm, which is only 7.7 times larger than that of the bulk copper. With no sign of copper observed outside the line area, it can be inferred that the copper can selectively grow on the laser patterned line zone rather than on the acceptor layer.

**Figure 4:**
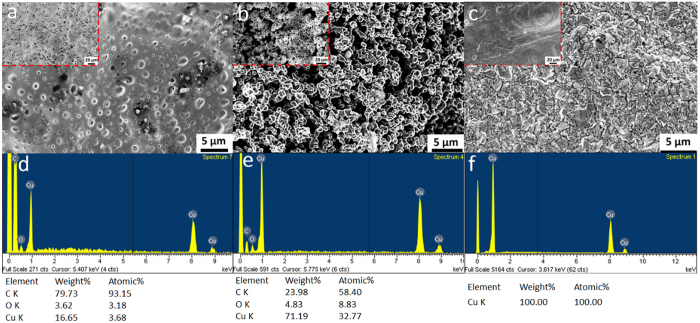
The SEM analysis of the CuO/ABS composite before and after LIFT. The SEM plan view images of (**a**) as-prepared CuO/ABS composite as donor layer, (**b**) LIFT patterned layer on acceptor layer, and (**c**) LIFT patterned layer after electroless plating. The insets in (**a**,**b**,**c**) are their corresponding zoom out images (**d**,**e**,**f**) are the EDX results of (**a**,**b**,**c**), respectively.

To explore the microstructures of the CuO/ABS composite before/after laser treatment and electroless-plating, the plan-view scanning electron microscopy (SEM) images are shown in Fig. 4(a–c), respectively. The as-prepared CuO/ABS composite is displayed as a dense film with micro-size clusters as represented in Fig. 4(a). The CuO/ABS composite surface is smooth as indicated in the inset of Fig. 4(a) over areas of several-mm^2^. After the LIFT, however, the composite surface becomes porous, as revealed in Fig. 4(b). According to the CuO/ABS composition, the clusters may be the aggregated particles of CuO and its photochemically reacted products of Cu, which can be confirmed in the following elementary characterizations in the next section. With zoom-out magnification, the micro-size clusters and holes are randomly distributed across the whole area, as shown in the inset of Fig. 4(b). The roughness of the as-transferred sample is 1069 nm as revealed by atomic force microscopy (AFM) (see Fig. S2(a)). The rough surface of the as-transferred sample is consistent with the darker bronzing color in Fig. 3(b). The highly porous structures could also explain the poor resistivity of the as-prepared sample. Finally, after electroless plating, the surface of the sample is smoother, as evidenced in Fig. 4(c). The roughness is reduced to 299 nm as determined by AFM, and shown in Fig. S2(b). Similar smoothness is also observed with a larger area, as in the inset of Fig. 4(c), which is consistent with the brighter optical microscope image in Fig. 3(c). It can be deduced that a thick copper layer grew on the laser transferred compositions, filling and sheathing the underlying porous structures.

The energy-dispersive X-ray spectroscopy (EDX) analysis results of the samples are depicted in Fig. 4(d–f). The as-prepared CuO/ABS composite is primarily determined to be carbon with an atomic ratio up to 93.15%, as shown in Fig. 4(d). The remaining characterized elements are mostly copper and oxygen with the atomic ratio close to 1:1. The main compositions of the as-prepared donor layer are thus the CuO and ABS materials as prepared. After LIFT of the donor layer onto the acceptor layer, the elementary compositions change, as shown in Fig. 4(e). The relative amount of oxygen to copper has a significant decrease from 1:1 to 1:4, suggesting remarkable deoxygenizing of CuO during laser treatment. Meanwhile, the amount of carbon also decreased substantially from 93.15% to 58.4%, suggesting that a huge amount of carbon is removed after the laser transfer process, as shown in Fig. 4(e). The active carbon content reacts with the air during the LIFT and leads to a weight loss of carbon. So the decomposition of ABS should be the dominant driving force for the LIFT ejection momentum. Lastly, after growing thick films during the electroless plating, the only element that could be observed after electroless-plating was Cu, as depicted in Fig. 4(f). Therefore, it can be deduced the electroless-plating process deposited thick Cu films above the selective laser transferred area, which is consistent with the resistivity improvement result previously measured.

**Figure 5:**
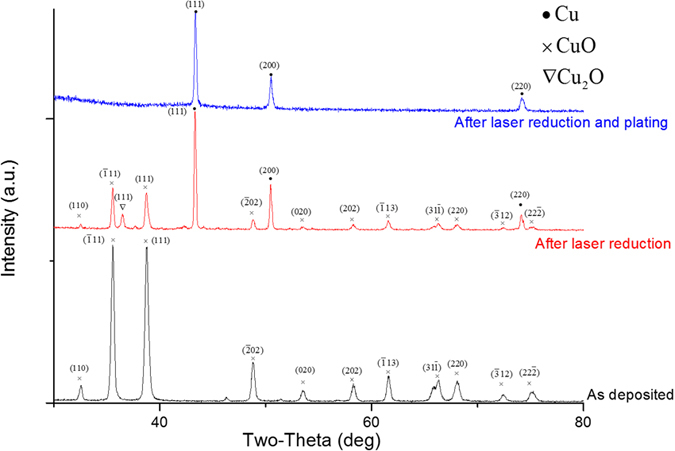
The XRD patterns of the LIFT Cu. From bottom up, the black, red and blue spectra are for the as-deposited CuO/ABS composite, the composite after laser treatment, and the composite after post electroless copper plating, respectively.

To further confirm the chemical components through the LIFT and post copper plating, X-ray diffraction (XRD) was performed, as shown in Fig. 5. The as-prepared CuO/ABS composite as a donor layer shows typical XRD patterns for monoclinic crystal copper(II) oxide as the black profile marked with the cross symbol^44^. Then, after laser treatment, the CuO identification peaks are still preserved with weaker intensities, marked as the red profile. Meanwhile, dominant face centred cubic phase Cu identification peaks are noted with dot symbols, suggesting the Cu can be synthesized after laser homolysis of CuO. In addition, a tiny face-centered cubic Cu_2_O (111) peak at 37 degrees was also observed, suggesting incomplete reduction of CuO also occurred. Thus the 405 nm laser can effectively reduce copper(II) oxide to copper(0) and copper(I) oxide.

A control of the laser wavelength is crucial for our photochemical LIFT method, rather than the laser power which is a key control parameter for traditional LIFT^15^,^34^,^45^,^46^. To corroborate the importance of the laser wavelength rather than the laser power, a control experiment was performed with a 10 W fiber laser at 1064 nm. Although the resulting heating effect can still decompose the ABS materials and leave behind the isolated copper oxide clusters as shown in Fig. S3, there was no copper synthesized, as proven by the XRD patterns in Fig. S4. From the bond dissociation energies shown in Table S1, it can be inferred the laser wavelength is more important for the laser energy absorption than the laser output power^47^ for the photochemical reduction of CuO. With a low power of 0.4 W, the 405 nm laser with 3.1 eV photon energy can still effectively reduce the copper(II) oxide to its unoxidized state copper(0), which proved to be impossible for a 1064 nm laser, even with a 20-fold increase of the laser power. Eventually, for the 405 nm laser treated sample after further electroless plating, only Cu identification peaks could be observed, as shown in the blue profile in Fig. 5. So the thick plated Cu layer covers the underlying composition and overwhelms their signals, which is consistent with the EDX results in Fig. 4(f), as well as the brighter color in Fig. 3(c).

**Figure 6:**
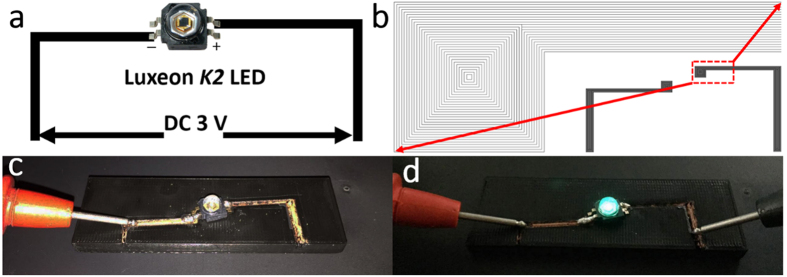
Prototypes of circuits for wiring up LEDs. (**a**) The circuit design for powering up a commercial LED. (**b**) The circuit pattern design for LIFT process. The magnification is the corresponding laser writing path. (**c**) The wired up circuit with LIFT built circuit on a PLA substrate for powering LED in an open circuit status. (**d**) The power up status of (**c**) and the green color LED is lighted on.

The above results for establishing the highly conductive copper line pattern can be successfully transferred from the 100 μm thick ABS/CuO donor layer by LIFT and post plating. In order to demonstrate the potential application of this technique, a simple LED interconnection was designed and built, as shown in Fig. 6. A high-power LED was connected to a 3 V direct-current (DC) power supply as shown in Fig. 6(a). The interconnection circuit was then divided into the laser writing paths shown in Fig. 6(b), so the laser beam followed the path and selectively transferred the donor layer onto the target layer, such as FR-4, polyimide, glass, and even 3D printed thermoplastic. As a demonstration, one 3D printed polylactic acid (PLA) substrate was used as an acceptor layer onto which the interconnection circuit was fabricated. Figure 6(c) shows the prototype built by additive manufacturing of copper circuits on a 3D printed PLA substrate. The black substrate is the 3D printed PLA built by a fused deposition modelling printer and the metal parts in yellow are the interconnection circuit lines. After the circuits were connected, the green LED was powered up by a 3 V DC power supply, as shown in Fig. 6(d).

**Figure 7:**
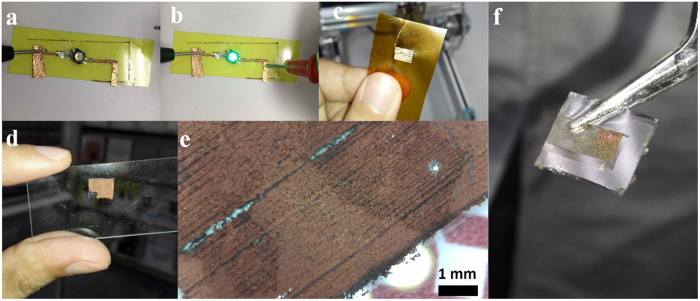
LIFT patterned copper on various substrates. (**a**) FR-4 laminate with LED off. (**b**) FR-4 laminate with LED on. (**c**) Polyimide. (**d**) Glass slide and (**e**) optical microscope close-up image. (**f**) PDMS substrate.

Apart from the PLA substrate, this LIFT method has also been successfully implemented onto other substrates, including FR-4 laminates, polyimide and glass slides, as shown in Fig. 7(a–e). However, patterning copper on the PDMS substrate is difficult with the current LIFT method. A typical defective sample is shown in Fig. 7(f), indicating that the copper cannot be fully patterned on the PDMS substrate as designed. The reason for the quality defects can be ascribed to the low surface free energy of the PDMS substrate. In order to compare the surface energies of different substrates used in this work, water contact angle measurement was undertaken for polyimide, glass slide, PDMS and FR-4 laminate substrates, as shown in Fig. S5. The glass slide had the lowest water contact angle of 37.3° among these 4 types of substrates. As a consequence, the success rate for patterning copper on glass slides is large as demonstrated in Fig. 7(d,e), due to its high surface energy. Nevertheless, the surface energies of polyimide and FR-4 laminate substrates are still sufficiently high with water contact angles of 76.4° and 66.2°, respectively, so patterning of copper on these laminates is still possible, as illustrated in Fig. 7(a–c). However, the surface energy of PDMS is relatively low, and PDMS behaves hydrophobically with a contact angle of 105.4°. So the quality defects when fully patterning copper onto PDMS, as in Fig. 7(f), should be due to the low surface energy, similar to other additive patterning methods, such as inkjet printing^48^. Further surface modification of PDMS for facilitating patterning of copper using this LIFT method can be improved by ozone plasma^49^ or chemical surfactant treatment^50^, methods which will be studied in the future work.

## Methods

### Computational Methods

Geometric optimization and optical calculations of copper(II) oxide are performed using spin polarized density functional theory (DFT) plus the effective Coulomb interaction (U) (DFT+U) formalism method implemented in the Vienna ab initio Simulation Package (VASP)^51^. The exchange-correlation energy is computed by using the Perdew-Burke-Ernzerhof (PBE) function, and the ion-electron interaction is solved with the projector-augment-wave technique^51^,^52^. For geometric optimization, both the lattice constants and atomic positions are relaxed until the forces on the atoms are less than 0.02 eV/Å and the total energy change is less than 1.0 × 10^−5^ eV. A 5 × 7 × 5 Monkhorst-Pack^53^ grid and a kinetic energy cutoff of 5 eV are selected with a U value of 7.12 eV^54^. For static and optical calculations, a finer 20 × 28 × 20 grid is chosen. The wavelength-dependent dielectric function is calculated, and then the absorption coefficient *α*(*E*) as a function of photon energy is evaluated according to the following expression Eq. (1):


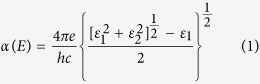


## Materials

Copper(II) oxide was purchased from the International Laboratory Co. (USA) and ABS (PA-707) was purchased from the Chi Mei Co. (Taiwan) as the sacrificial components which provide the kinetic energy for the LIFT process^55^. 1 mol CuO and 1 mol ABS were added and dissolved into the chloroform solution, all as purchased, and the composite was stirred for 1 h using hot plate heating at 60 °C. The composite was then brush-painted onto glass slides and doctor-bladed to a thickness of 100 μm, after which it was heated on a hot plate at 60 °C for 1 h. The electroless plating solution ECM-110 was purchased from *Teamly Chemicals Corp.* and contained CuSO_4_ and edetic acid with a ratio of 16:5. The laser transfer patterned samples were dipped in the ECM-110 solution at 60 °C for 4 h.

### Laser writing system

A simple laser writing system was constructed and is shown in Fig. 4(a). A 405 nm GaN laser with output power fixed at 0.4 W was mounted on a tailor-made X-Y moving system driven by 2 stepper motors. The circuit patterns were designed with Autodesk AutoCAD software, with each line representing the width at 50 μm. The CAD files were transformed to G-code using open-source GRBL firmware and loaded to an open-source Arduino microcontroller. The speed of the laser writing was fixed at 10 mm/min. The transparent slides with the CuO/ABS composite coating were placed above the acceptor layer with the CuO/ABS composite side facing down. Scotch tape of 50 μm thickness was inserted as a spacer between the donor and acceptor layers. The laser beam was focused onto the donor layer and the laser beam size was measured to be 200 μm.

### Characterization

The optical microscopy images were captured with a Leica Microscope using a 50x object lens and CCD. The microstructures were analyzed with a LEO 1530 Field Emission Scanning Electron Microscopy (FESEM) with an Energy Dispersive X-ray Spectrometer (EDX). The crystalline structures were studied with a Bruker D8 Advanced X-ray Diffractometer (XRD) system. The roughness of the samples was measured with Bruker NanoScope 8 scanning probe microscopy at the tapping mode. The resistivity of the sample circuits was determined by standard four-probe measurement using an Agilent 4338B milliohm meter.

## Conclusions

In summary, a novel additive manufacturing platform is proposed and demonstrated for building highly conductive copper circuit lines using simultaneous LIFT and photochemical reduction with a 0.4 W 405 nm laser. DFT calculations showed that the light absorption coefficients of CuO at 405 nm were sufficiently high for triggering photochemical homolysis. Exhibiting very high rates of photon absorption and thermal expansion, the CuO/ABS donor films can be directly transferred onto an arbitrary acceptor layer using a relatively cheap laser. Copper was directly synthesized after 405 nm laser reduction, achieving a resistivity of 1.46 × 10^−6^ Ωm. Post electroless-plating can selectively coat the laser patterned area and form thick copper films onto the patterned lines, with resistivity down to 1.29 × 10^−7^ Ωm. The photochemical reduction products were confirmed to be dominated with copper by the EDX and XRD results. This platform opens up a novel area for applying the LIFT process as a low-cost and high throughout technique for 3D printing of metallic circuits onto various types of substrates. This novel LIFT method is envisioned to have a wide range of vital applications, such as biomedical sensors^48^, molded interconnect devices^56^, 3D micro batteries^57^, and microfluidics^58^. In addition to copper, the other metals shown in Table S1 might also be printed by this novel platform which will be studied in future research, with the laser source having the appropriate wavelength. This LIFT method is effective and efficient for patterning interconnections compared to other additive methods. For example, the inkjet printing of copper interconnections requires multiple runs of both printing and sintering under an inert gas environment in order to achieve low resistivity^59^. The selective laser sintering method requires a relatively high working temperature (890°) and a high laser power (100 W)^60^. In addition, this open source laser system setup is more cost-effective compared to other additive methods using expensive instruments. Clearly, this novel LIFT method has advantages in printing metal interconnections due to the simplicity of the working conditions and lower process expenses. The ejection mechanism of continuous-wave LIFT may be similar to nanosecond-LIFT, since the film reaches a local equilibrium over its thickness^45^. In nanosecond-LIFT, thermal expansion of the film and local evaporation are known driving mechanisms. However, future work will be performed to conclusively describe the herein observed ejection^46^.

## Additional Information

**How to cite this article**: Yung, W. K. C. *et al*. Additive and Photochemical Manufacturing of Copper. *Sci. Rep.* **6**, 39584; doi: 10.1038/srep39584 (2016).

**Publisher's note:** Springer Nature remains neutral with regard to jurisdictional claims in published maps and institutional affiliations.

## Supplementary Material

Supplementary Information
