# Supplementary material for: Additive and Photochemical Manufacturing of Copper
Source: Sci Rep. 2016 Dec 21;6:39584. doi: 10.1038/srep39584 (PMC5175138; doi:10.1038/srep39584)
Supplement: Supplementary Information [file srep39584-s1.doc]

# Supplementary Information

Additive and Photochemical Manufacturing of Copper

Winco K. C. Yung^1^†, Bo Sun ^1,2^†, Zhengong Meng^3^†, Junfeng Huang^1^†, Yingdi Jin^4^†, Hang Shan Choy^1^, Zhixiang Cai^1^, Guijun Li^1^*, Cheuk Lam Ho^3^*, Jinlong Yang^4^, Wai Yeung Wong^3,5^*

^1^Department of Industrial and Systems Engineering, The Hong Kong Polytechnic University, Hung Hom, Hong Kong, HKSAR. Email: [mitch.li@polyu.edu.hk](mailto:mitch.li@polyu.edu.hk)

^2^School of Reliability and Systems Engineering, Beihang University, No. 37 Xueyuan RD. Haidian, Beijing 100191, China.

^3^Institute of Molecular Functional Materials and Department of Chemistry, The Hong Kong Baptist University, Waterloo Road, Hong Kong, HKSAR. Email: [clamho@hkbu.edu.hk](mailto:clamho@hkbu.edu.hk)

^4^Hefei National Laboratory for Physical Sciences at Microscale, University of Science and Technology of China, Hefei, Anhui 230026, China

^5^Department of Applied Biology and Chemical Technology, The Hong Kong Polytechnic University, Hung Hom, Hong Kong, HKSAR. Email: [wai-yeung.wong@polyu.edu.hk](mailto:wai-yeung.wong@polyu.edu.hk)

† These authors contributed equally.


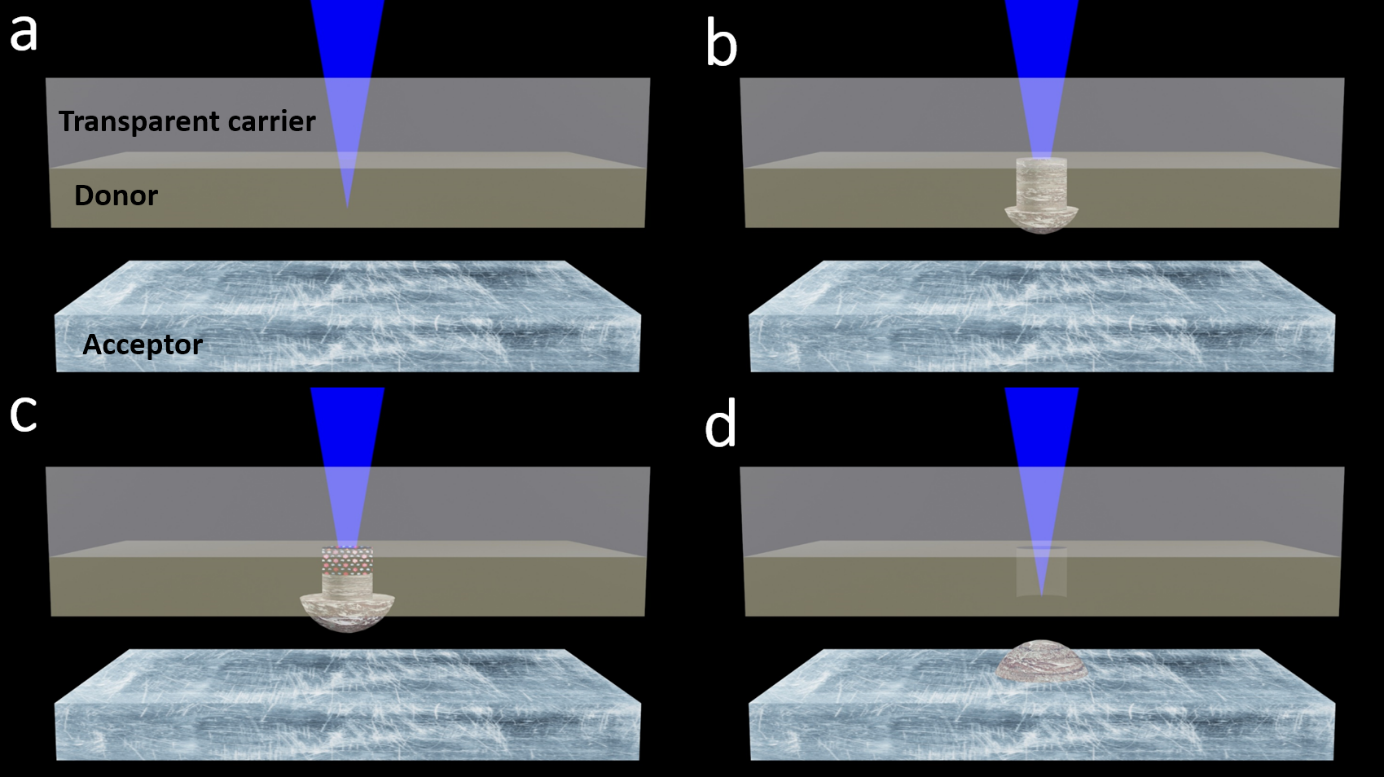


**Figure S1. Standard laser induced forward transfer process with pulsed laser.** (**a**) The multi-layer outline during LIFT patterning. (**b**) The selective spot after laser illumination expands in size. (**c**) Further volume expansion occurs when the gas is exhausted from the decomposition of the donor material. (**d**) The selective droplets are ejected and coated onto the acceptor layer.


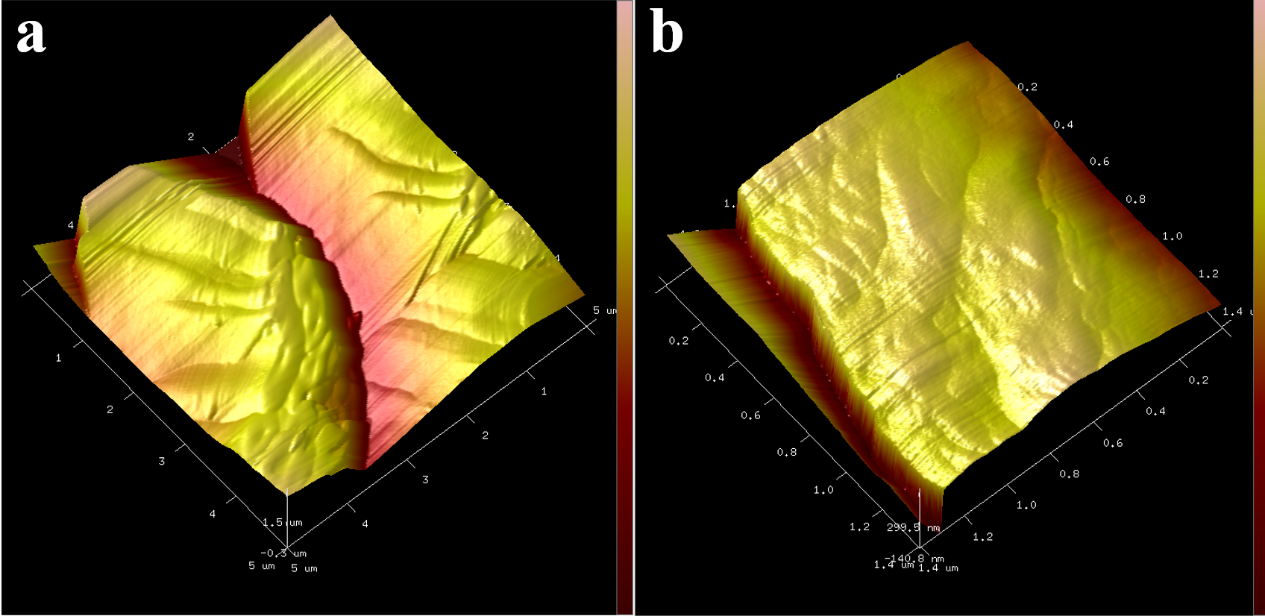


**Figure S2. Tapping mode AFM topologies.** (**a**) before and (**b**) after electroless copper plating.


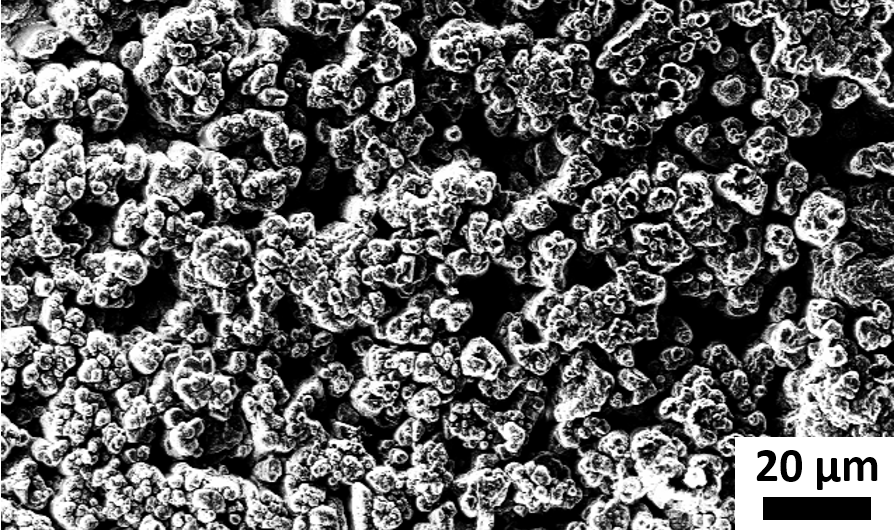


**Figure S3. SEM plan view images of the CuO/ABS composite after 10 W 1064 nm laser treatment.**


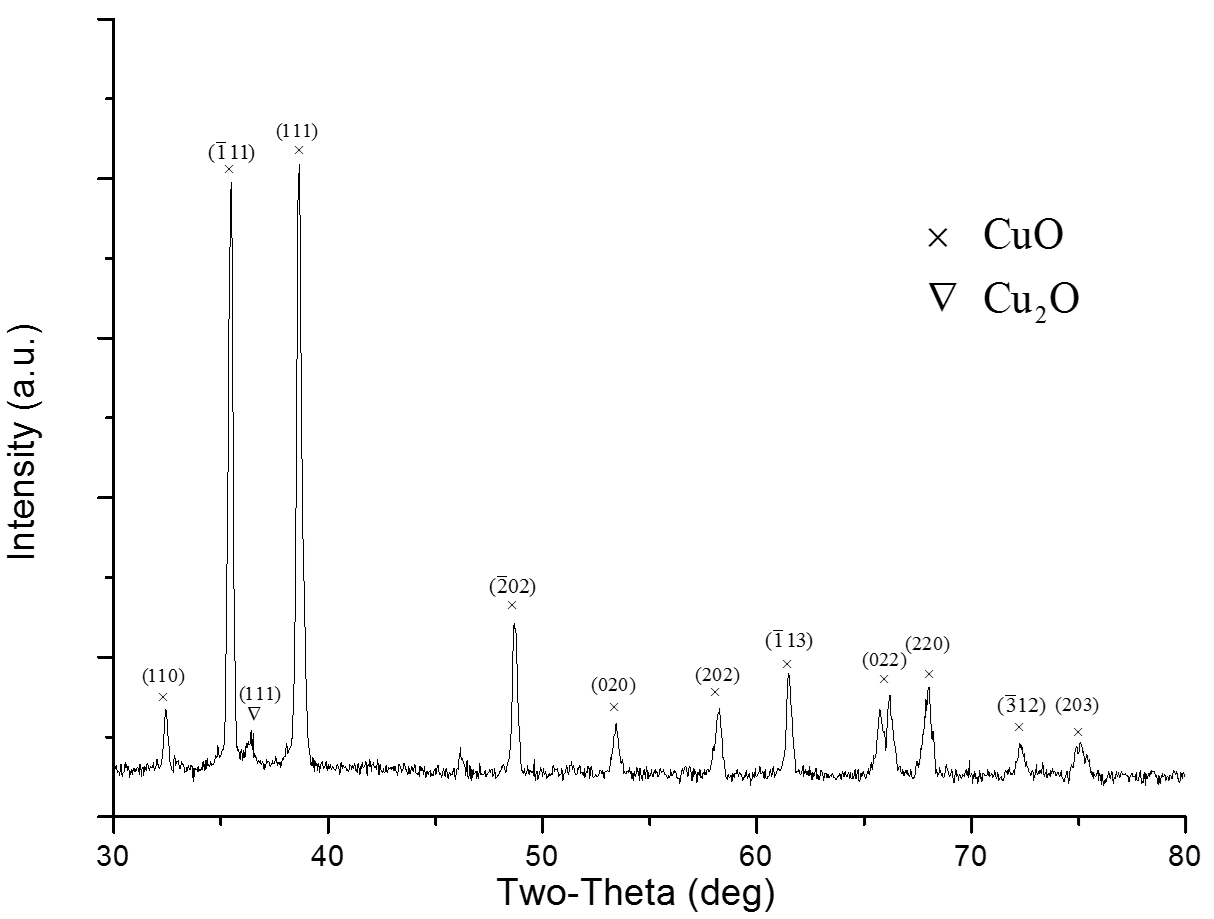


**Figure S4. XRD patterns of the CuO/ABS composite after the 10 W 1064 nm laser treatment.**


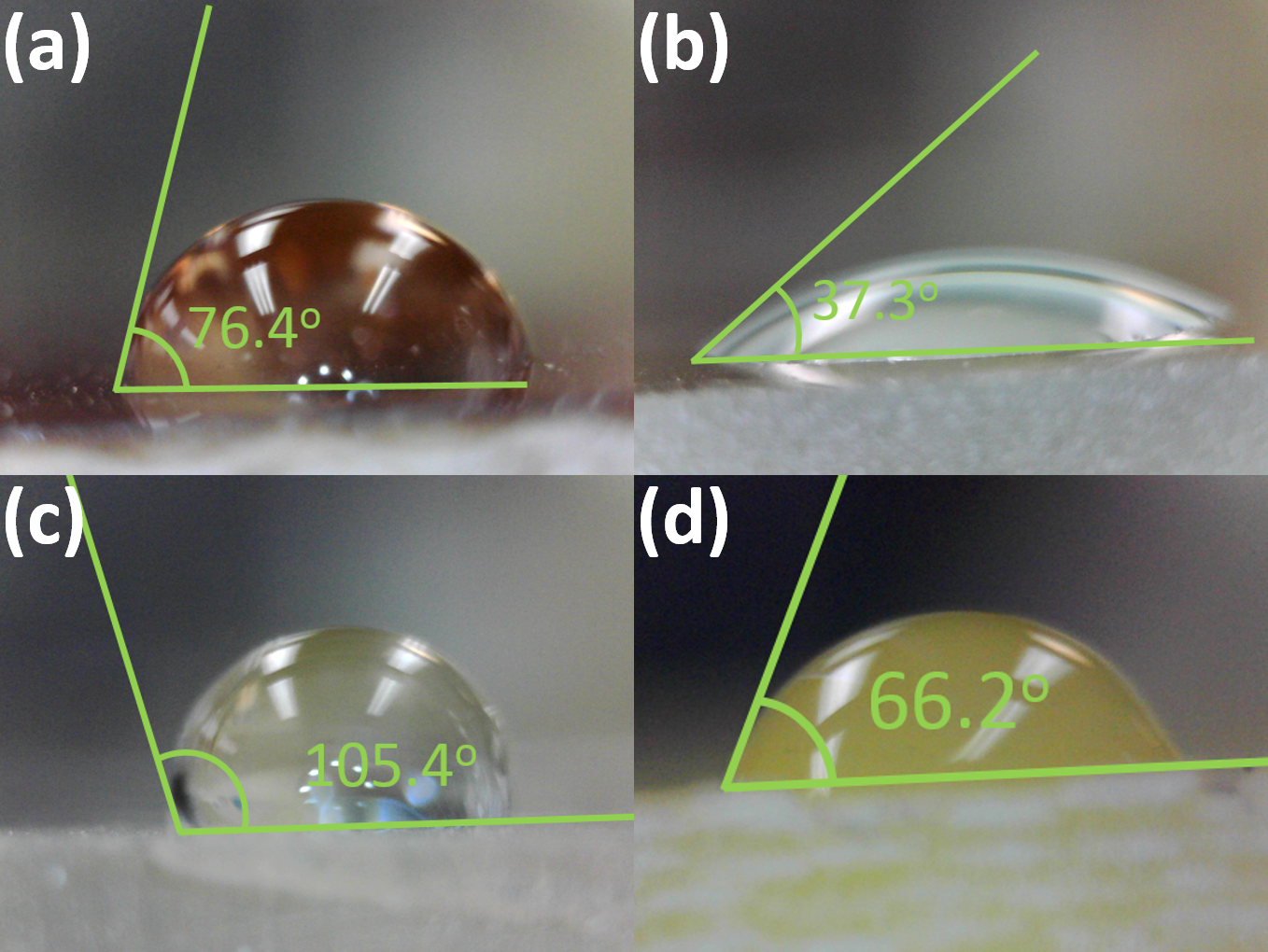


**Figure S5.** **Contact angle measurement for different substrates.** (**a**) Polyimide. (**b**) Glass slide. (**c**) PDMS. (d) FR-4 laminate.

| **Element** | **Resistivity (Ω·m) (20 ^o^C)** | **Oxide Bond** | **Bond Dissociation Energies (kJ mol^-1^)** |
| --- | --- | --- | --- |
| Silver | 1.59×10^−8^ | Ag-O | 221 ± 21 |
| Copper | 1.68×10^−8^ | Cu-O | 287.4 ± 11.6 |
| Gold | 2.44×10^−8^ | Au-O | 223 ± 21 |
| Alumina | 2.82×10^−8^ | Al-O | 501.9 ± 10.6 |
| Cobalt | 6.24×10^−8^ | Co−O | 397.4 ± 8.7 |
| Nickel | 6.99×10^−8^ | Ni−O | 366 ± 30 |
| Iron | 1.00×10^−7^ | Fe-O | 407.0 ± 1.0 |
| Platinum | 1.06×10^−7^ | Pt-O | 418.6 ± 11.6 |
| Titanium | 4.20×10^−7^ | Ti-O | 666.5 ± 5.6 |

**Table S1.** **Resistivity of the metals and the bond dissociation energies of their corresponding oxides.**
